# Estimation of Thermodynamic Stability of Methane and Carbon Dioxide Hydrates in the Presence of Hydrogen Sulfide

**DOI:** 10.1021/acsomega.2c02823

**Published:** 2023-02-07

**Authors:** Jai Krishna Sahith Sayani, Niall J. English, Muhammad Saad Khan, Bhajan Lal, Venkateswara Rao Kamireddi

**Affiliations:** † School of Chemical and Bioprocess Engineering, University College Dublin, Belfield D04 V1W8, Dublin, Ireland; ‡ CO2 Research Center (CO2RES), Universiti Teknologi PETRONAS, Bandar Seri Iskandar 32610, Perak, Malaysia; § Chemical Engineering Department, Universiti Teknologi PETRONAS, Bandar Seri Iskandar 32610, Perak, Malaysia; ∥ Department of Petroleum Engineering & Petrochemical Engineering, University College of Engineering (A), Jawaharlal Nehru Technological University—Kakinda, Kakinda 533003, India

## Abstract

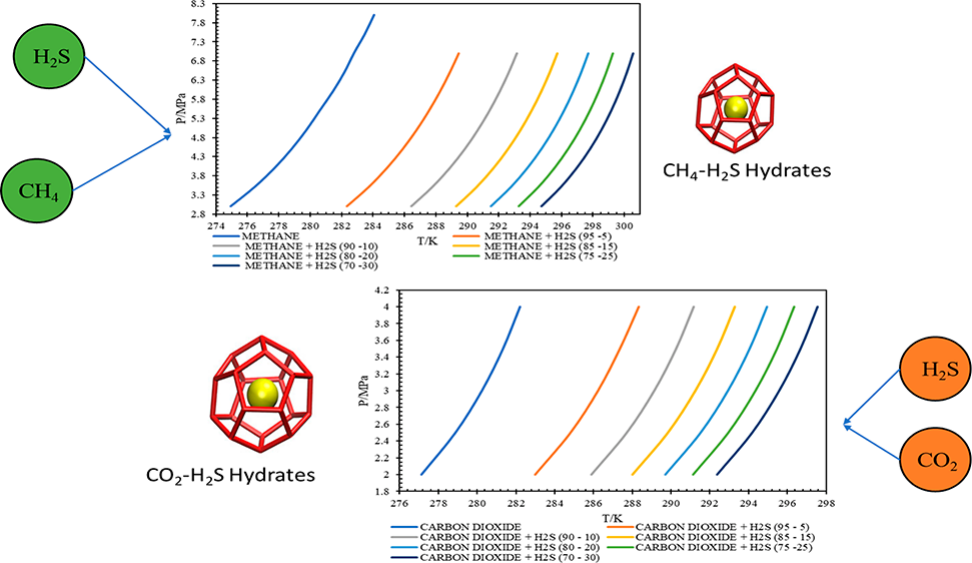

This work presents the effect of hydrogen sulfide gas on the phase behavior of both methane gas hydrate formation and CO_2_ gas hydrate formation. For this, the thermodynamic equilibrium conditions for various gas mixtures containing CH_4_/H_2_S and CO_2_/H_2_S are initially found by simulation using PVTSim software. These simulated results are compared using an experimental approach and the available literature. Then, the thermodynamic equilibrium conditions generated by simulation are used for generating Hydrate Liquid–Vapor-Equilibrium (HLVE) curves to understand the phase behavior of gases. Further, the effect of hydrogen sulfide on the thermodynamic stability of methane and carbon dioxide hydrates was studied. It was clearly observed from the results that an increase in H_2_S composition in the gas mixture decreases the stability of CH_4_ and CO_2_ hydrates.

## 1 Introduction

Gas hydrates are solid, crystal-like compounds formed when guest molecules are trapped within a cage-like structure made of water molecules.^1,2^ The guest molecules that are commonly formed into gas hydrates are Methane (CH_4_), Ethane (C_2_H_6_), Propane (C_3_H_8_), Nitrogen (N_2_), Carbon dioxide (CO_2_), and Hydrogen sulfide (H_2_S).^3,4^ Gas hydrates are part of the clathrate hydrate group, widely spread in nature. The water molecules are referred to as the host molecules, and the guest molecules are the gas contained within the host molecules. The crystals are stabilized with the result of van der Waals forces, where no bonding occurs between the host and guest molecules as the guest molecules can freely rotate in the water cage.^5−7^

Out of all the hydrate formers, Hydrogen sulfide (H_2_S) is highly toxic and an aggressive hydrate former.^8,9^ Being a slightly polar molecule, H_2_S exhibits a unique effect of dipole moment for hydrate stabilization.^10,11^ Hydrogen sulfide (H_2_S) stabilizes both small and large cavities of structure-I hydrates.^12,13^ Oil or natural gas is considered sour if it has a high percentage of hydrogen sulfide. According to the Texas Commission on Environmental Quality, natural gas containing more than 20 ppm H_2_S by volume is generally considered a sour gas.^14,15^ In addition to being toxic, hydrogen sulfide in the presence of water also damages piping and other equipment handling sour gas by sulfide stress cracking. H_2_S acidifies the water, which causes pitting corrosion to carbon steel pipelines. Corrosion reaction increases fast when it combines oxygen and carbon dioxide (CO_2_).^16,17^ Thus, they can significantly reduce the service life of transportation pipelines and processing facilities in the oil and gas industries. Natural gas that contains any of the acid gases like carbon dioxide or hydrogen sulfide is termed an acid gas.^15,18^

In deep-water pipelines, the elimination of natural gas hydrates is prominent as their formation can pose a threat to both the economy and safety. Besides that, the presence of H_2_S in gas hydrates will aggravate the risk. Research in gas hydrates is getting more intensive. Hydrate properties, formation and dissociation conditions, and effective means of hydrate removal have all been studied thoroughly.^19^

Therefore, this work presents the effect of hydrogen sulfide gas on the phase behavior of both methane gas hydrate formation and CO_2_ gas hydrate formation. For this, the thermodynamic equilibrium conditions for various gas mixtures containing CH_4_/H_2_S and CO_2_/H_2_S are initially found by simulation using PVTSim software. These simulated results are compared using the experimental approach and the available literature. Then, the thermodynamic equilibrium conditions generated by simulation are used for generating HLVE curves to understand the phase behavior of gases. Further, the effect of hydrogen sulfide on the thermodynamic stability of methane and carbon dioxide hydrates was studied.

## 2 Methodology

### 2.1 Materials

The list of materials used to compare the simulation data by the experimental investigation of the formation of gas hydrates is presented in Table 1 and Table 2.

**Table 1:** Materials and Sources for Methane and Hydrogen Sulfide Gas Mixtures

S. No.	Material	Purity	Source
1	Methane Gas (CH_4_)	99.97%	Air Product Sdn. Bhd.
3	Mixed Gas-1	(CH_4_, 95%; H_2_S, 5%)	
4	Mixed Gas-2	(CH_4_, 90%; H_2_S, 10%)	
5	Mixed Gas-3	(CH_4_, 85%; H_2_S, 15%)	
6	Mixed Gas-4	(CH_4_, 80%; H_2_S, 20%)	
7	Mixed Gas-5	(CH_4_, 75%; H_2_S −25%)	
8	Mixed Gas-6	(CH_4_, 70%; H_2_S, 30%)	
9	Deionized Water	N/A	Gas Hydrate Research Laboratory, UTP

**Table 2:** Materials and Sources for Carbon Dioxide and Hydrogen Sulfide Gas Mixtures

S. No.	Material	Purity	Source
1	Carbon Dioxide (CO_2_)	99.97%	Air Product Sdn. Bhd.
3	Mixed Gas-1	(CO_2_, 95%; H_2_S, 5%)	
4	Mixed Gas-2	(CO_2_, 90%; H_2_S, 10%)	
5	Mixed Gas-3	(CO_2_, 85%; H_2_S, 15%)	
6	Mixed Gas-4	(CO_2_, 80%; H_2_S, 20%)	
7	Mixed Gas-5	(CO_2_, 75%; H_2_S, 25%)	
8	Mixed Gas-6	(CO_2_, 70%; H_2_S, 30%)	
9	Deionized Water	N/A	Gas Hydrate Research Laboratory, UTP

### 2.2 Experimental Apparatus

**Figure 1:**
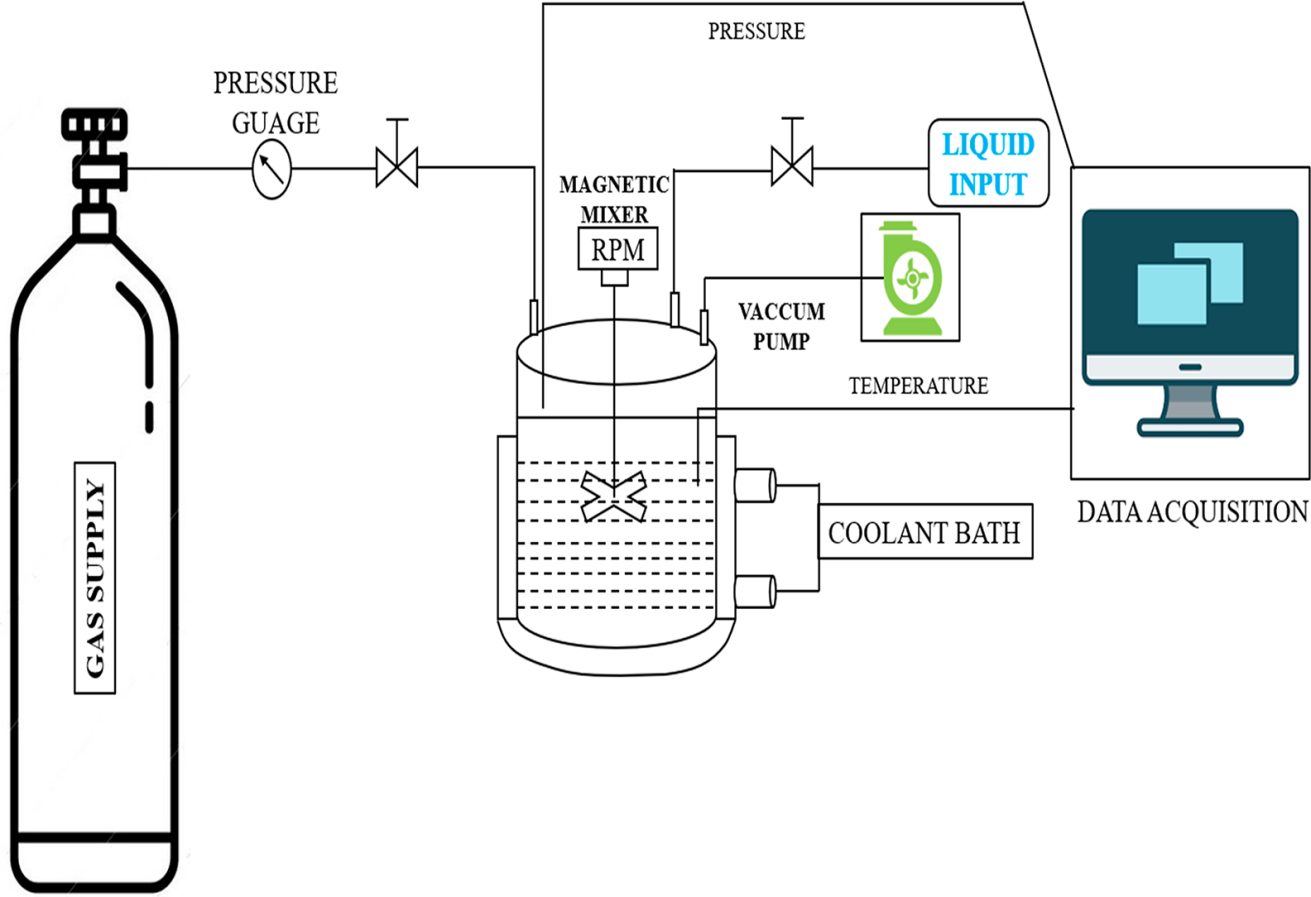
Schematic diagram of the experimental setup.

The schematic representation of the experimental setup used in this work is shown in Figure 1. Phase behavior of gas hydrates is evaluated with a device fitted with a 700 mL stainless steel high-pressure reactor. The temperature range of the reactor is −20 to 40 °C, and the pressure limit for the reactor is 20 MPa. Pressure and temperature sensors connected to a data-logging device in the reactor are used to determine the pressure and temperature changes. The time span for the recording was maintained as 10 s. A 4-bladed impeller magnetic stirrer is positioned inside the reactor to provide adequate agitation during the hydrate test. Device temperature is controlled by a thermostatic bath fitted with a PID controller at an accuracy of ±0.3 °C.

### 2.3 Experimental Procedure

Thermodynamic equilibrium conditions are determined by the T-cycle method with isochoric slow-step heating. This experimental procedure is adapted from the literature.^20−23^ Prior to the experimental study, any impurities in the reactor cell were removed by washing it with distilled water and drying it thoroughly. A deionized water sample of volume of 200 mL is filled into the reactor cell to attain the pure system. Then the steadiness of the system reactor is maintained by cooling the cell embedded into the equipment to the selected working temperature. The temperature in the reactor cell is stabilized by pumping a small amount of gas using a high-pressure boosting pump. The vacuum in the reactor cell is created by releasing the gas pumped and operating the vacuum pump attached to the reactor cell. Then the gas flowed into the reactor cell up to the anticipated pressure after reaching the distinct temperature state.

Once stable temperature and pressure conditions are achieved, the magnetic stirrer is set at 400 rpm to interrupt the gas–liquid boundary interface during the formation of gas hydrates. The formation of gas hydrates was initiated by lowering the reactor cell temperature using quick cooling. The reactor was retained under similar conditions for an all-encompassing period of time after the desired temperature was reached (varies from 4 to 8 h). The formation of gas hydrate is an exothermic reaction; thus, a rapid drop in pressure with an increase in temperature in the data logging system is observed during gas hydrate formation. Once the hydrate formation is completed, no further drop in pressure is observed. Then the reactor is heated slowly with a stepwise rate of 0.5 K/h until the gas hydrate is fully dissociated.

Further, the equilibrium point of the hydrate is determined by maintaining the duration of each phase between 2 and 6 h. It took approximately 48 h to achieve each experiment study for hydrate formation and dissociation. Every experiment is repeated 3 times to ensure the elimination of uncertainty in the experiments, and the values presented are the average of the 3 experimental results.

### 2.4 Simulation Using PVTSim

65 data points of thermodynamic equilibrium conditions for various methane and hydrogen sulfide gas mixtures and 35 data points of thermodynamic equilibrium conditions for various carbon dioxide and hydrogen sulfide gas mixtures are found by simulation using PVTsim software. For the parameter of a cubic equation of state, PVTsim supports two distinct mixing rules. The most basic is the classical mixing rule, which requires, in addition to the above pure component values, a binary interaction parameter (*k*_*ij*_), which can be made temperature dependent and is used for nonpolar mixtures. The classical mixing rule is no longer adequate to represent the more complex polar–nonpolar interactions in the presence of brine (water + salt). PVTsim’s default polar component model is the Huron–Vidal (HV) mixing rule. It is based on a GE model that is Non-Random Two-Liquid (NRTL).^24−27^ However, because all of the analyzed gases and mixes are nonpolar, only the classical mixing rule is valid in this study. In addition, the SRK Peneloux equation of state was employed to predict phase behavior. The Peneloux method was chosen because it performs regular calculations to determine the precise gas gravities of the mixtures.

## 3 Results and Discussion

### 3.1 Comparisons of Simulated Data

**Figure 2:**
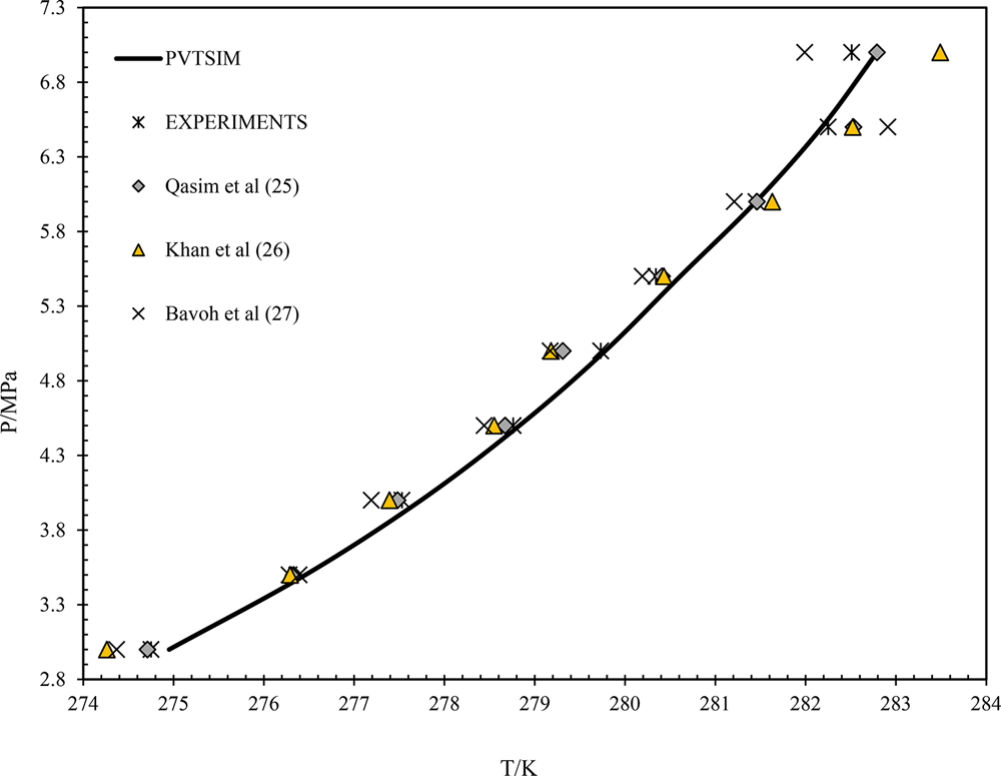
Pressure vs temperature graph of pure methane gas. Data from experiments and from refs (25−27).

**Figure 3:**
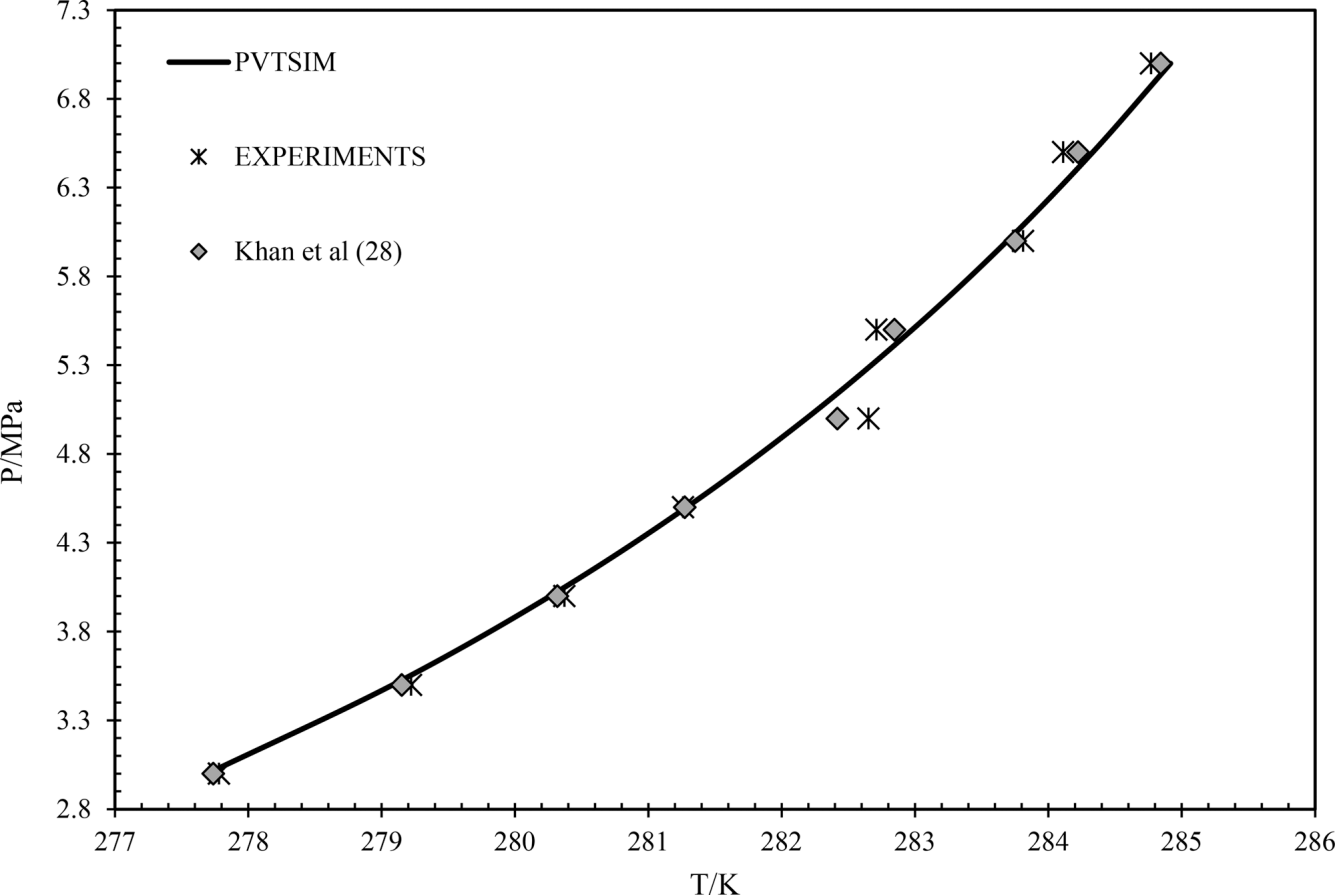
Pressure vs temperature graph of 70% methane and 30% carbon dioxide gas mixture. Data from experiments and from ref (28).

The data obtained from the PVTSim simulations are compared with experimental and literature-reported data. Since evaluating every condition through experiments is critical, some conditions are chosen randomly and evaluated. The data generated from the simulation, literature, and experiments are compared by plotting the *P* vs *T* graph. The comparison graph of the pure methane gas is shown in Figure 2.^28−30^ From Figure 2, it can be observed that the simulated data are in line with the experiments and the reported literature data. Similarly, the data of CH_4_–CO_2_ with 70–30 is presented^31^ in Figure 3. It can be observed that in all the cases, the simulated data are in accordance with the reported literature data, i.e., experimental data. This confirms the reliability and accuracy of the simulated data to the actual system during pipeline operations.

**Figure 4:**
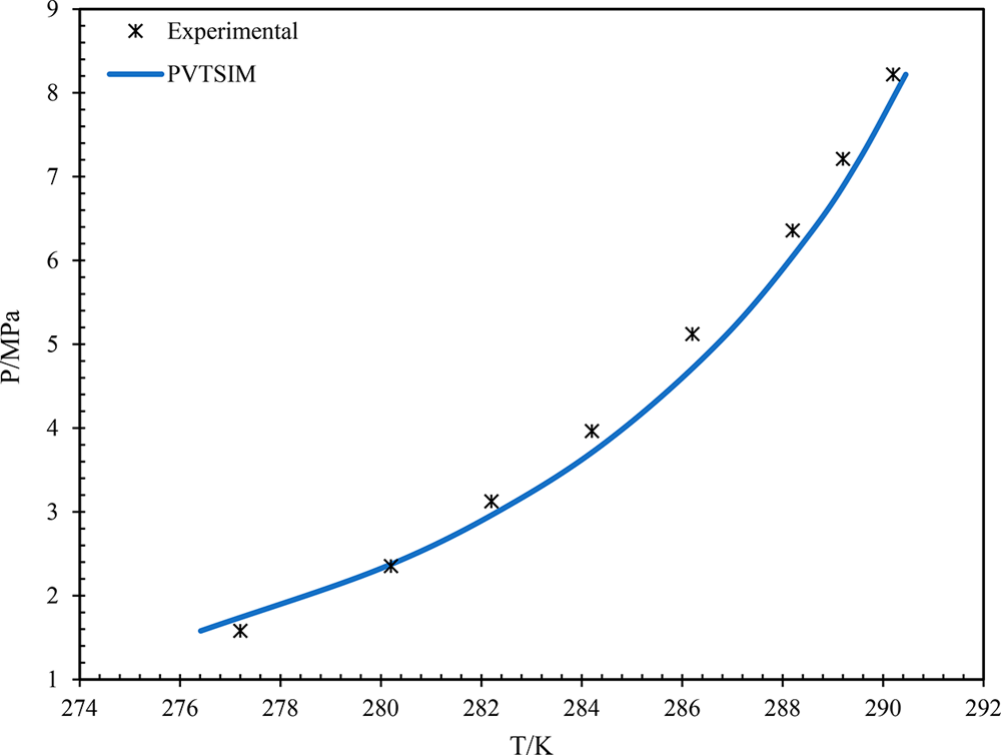
Experimental vs PVTSim data of gas-1.

**Figure 5:**
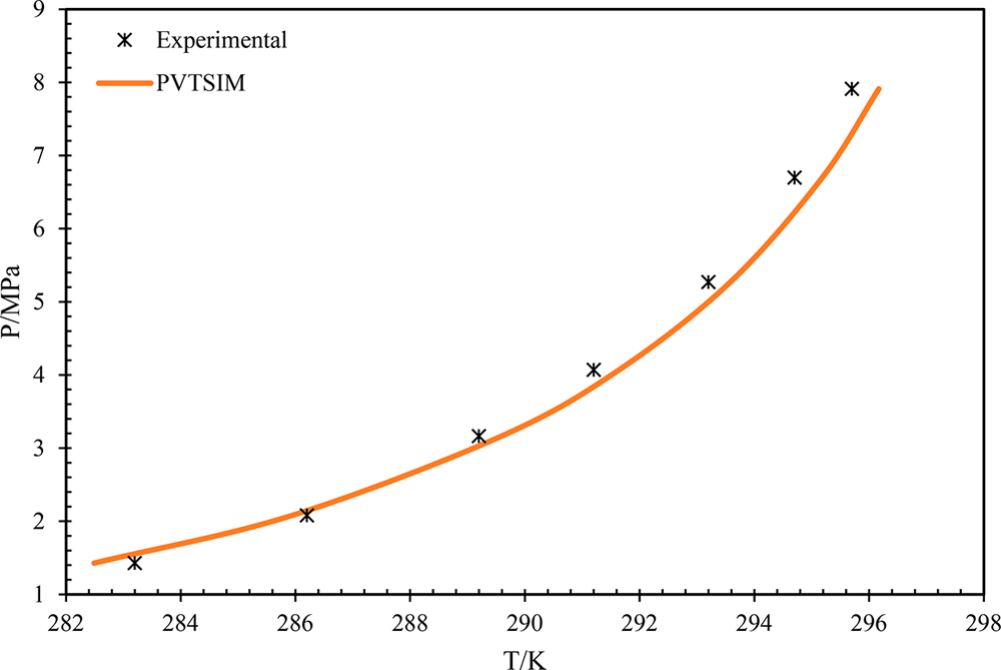
Experimental vs PVTSim data of gas-2.

Various literature data are available on different combinations of CH_4_/H_2_S/CO_2_. These were reported as experimental results. So, the data are collected and simulated in PVTSim to observe the error between the simulated and experimental data. The experimental data for 2 different gas combinations are captured from the literature.^32^ The gas mixtures considered were 87.65% CH_4_ + 7.40% CO_2_ + 4.95% H_2_S (Gas-1) and 77.71% CH_4_ + 7.31% CO_2_ + 14.98% H_2_S (Gas-2). The comparison plots are presented in Figure 4 and Figure 5.

### 3.2 Data Generation Using PVTSim Simulation

The PVTSim software package of the 2011 version is used for the data generation of hydrate formation conditions. SRK Peneloux equation of state was used in PVTsim software to determine the hydrate formation conditions. The equation is as follows:
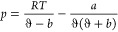
where *a* and *b* are EOS model parameters. The pure component parameters can be calculated by
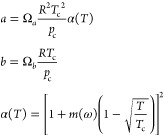
where
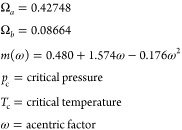


Seven gas samples of different combinations of CO_2_ and H_2_S are considered for data generation. Similarly, 7 other gas samples of different combinations of CH_4_ and H_2_S are considered. The simulation data obtained are presented in the Appendix.

### 3.3 Phase Behavior Analysis

**Figure 6:**
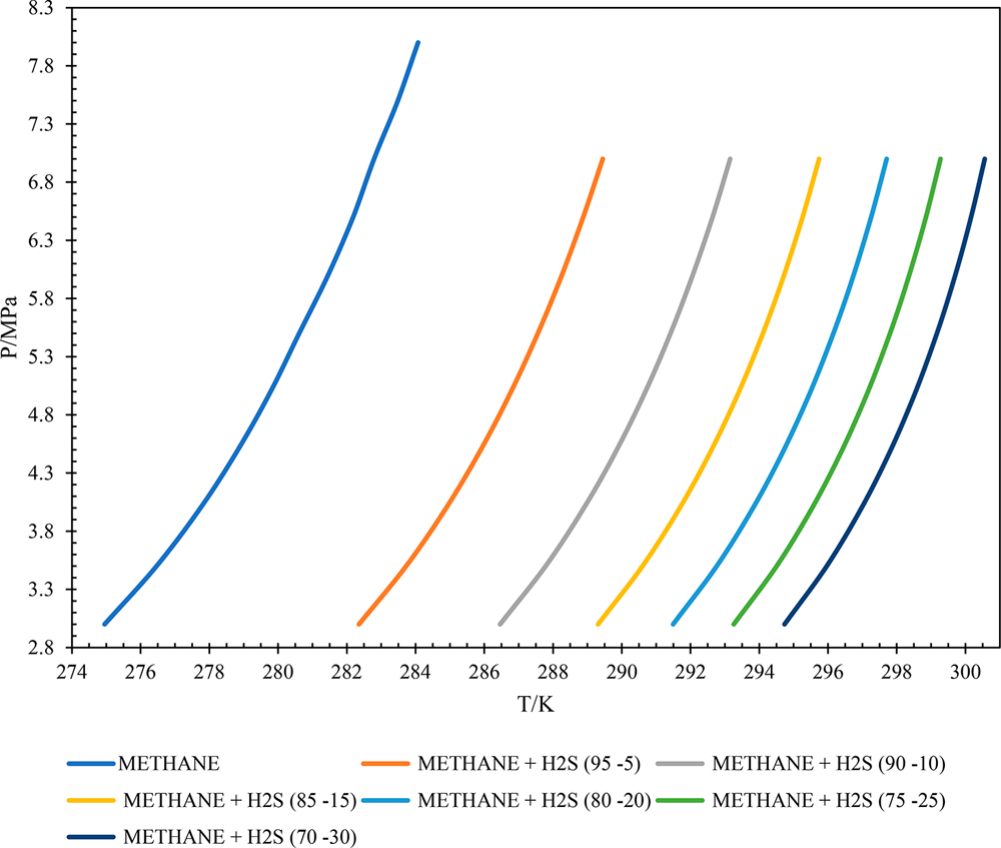
HLVE curves for methane and hydrogen sulfide gas mixtures.

The data obtained from PVTSim simulations are used to develop HLVE curves to understand the phase behavior of each gas. The HLVE plots for the considered methane and hydrogen sulfide gas mixture are presented in Figure 6. It can be clearly analyzed that, with the increase in the composition of H_2_S in the gas system, the HLVE curve shifts away from the pure methane line. So, with the increase in H_2_S content in the gas at a given pressure condition, the thermodynamic equilibrium temperature increases. From this observation, it can be concluded that the increase in H_2_S content in the gas system promotes gas hydrate formation.

**Figure 7:**
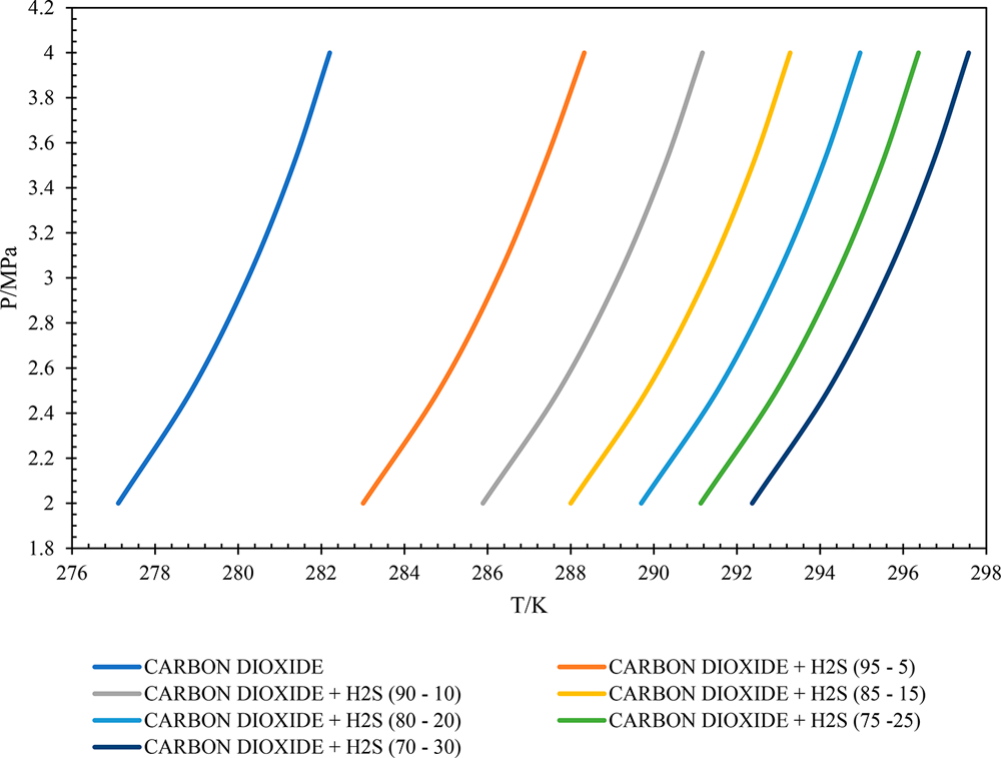
HLVE curves for carbon dioxide and hydrogen sulfide gas mixtures.

Similarly, the HLVE plots for the considered carbon dioxide and hydrogen sulfide gas mixtures are presented in Figure 7. It can be visualized from the HLVE curves that, with the increase in the composition of H_2_S in the gas system, the HLVE curve shifts away from the pure carbon dioxide line. So, the thermodynamic equilibrium temperature increases with the increase in H_2_S content in the gas at a given pressure condition. From this observation, it can be concluded that the increase in H_2_S content in the gas system promotes gas hydrate formation. This is due to the difference in molecular sizes of the methane, carbon dioxide, and hydrogen sulfide gases. When the hydrate conditions are favorable for formation, the smaller molecules tend to enter the water cages compared to larger gas molecules.^33,34^ So, the H_2_S hydrates form quicker and easier than CH_4_ and CO_2_. Unlike nonpolar hydrate formers like methane, hydrogen sulfide is a slightly polar molecule. Due to this, it shows a unique effect of dipole moment for hydrate stabilization.

It can be observed from Figure 7 that in the CO_2_–H_2_S mixture with 95–5 (mol %) composition, at higher pressure, the system shows a deviation and tends to move toward the pure CO_2_ gas. But this is not observed for CH_4_–H_2_S mixtures. This phenomenon is called the pseudoretrograde phenomenon. The pseudoretrograde phenomena will occur in any pseudobinary system where sI and sII formers with low vapor pressures are present.^35−37^ In the ternary system of CO_2_ + H_2_S + H_2_O, it is believed that sII hydrate will be formed. However, it should be noted that carbon dioxide also acts as an inhibitor to sII hydrate formation due to competition with H_2_S to occupy the large cages in the structure. Since carbon dioxide hydrate will be more stable in the form of sI hydrate, a structural transition will occur from sII into sI hydrate in the system. This structural transformation from sII to sI hydrate is believed to lead to the pseudoretrograde behavior in the system.

## 4 Conclusion

This work presents the effect of hydrogen sulfide gas on the phase behavior of both methane gas hydrate formation and CO_2_ gas hydrate formation. Various gas mixtures containing CH_4_/H_2_S and CO_2_/H_2_S are simulated in PVTSim software to predict the thermodynamic equilibrium conditions. The reliability of the software was verified before with data available in literature and experiments. As the H_2_S gas is highly toxic and extremely difficult to contain during experiments, the simulations are adapted in this study. In both situations, it was observed that the increase in H_2_S composition resulted in promotion behavior. As presented, when the H_2_S concentration increases in the system, the binary systems tend to move away from the HLVE curve of the pure CH_4_ or CO_2_ systems. From this, it can be concluded that the increase in H_2_S composition in the gas mixture decreases the stability of CH_4_ and CO_2_ hydrates as the dissociation temperature at a given pressure condition is higher than that of the pure systems. This study proves the importance of an accurate prediction process for H_2_S hydrate and an effective chemical inhibition technique. Also, this work helps as a fundamental study for studying contaminant separation from gases using gas hydrate technology.

## Appendix

Simulation data are provided in Tables 3 and 4.

**Table 3:** Data Points Generated by Simulation Using PVTSim Software for Methane Gas and Hydrogen Sulfide Gas Mixtures

Gas Sample	Gas Gravity	Pressure (MPa)	Temperature (K)
Methane	0.554	3	274.95
		3.5	276.46
		4	277.74
		4.5	278.83
		5	279.78
		5.5	280.59
		6	281.45
		6.5	282.18
		7	282.79
		7.5	283.48
		8	284.07
Methane + H_2_S (95–5)	0.585	3	282.35
		3.5	283.73
		4	284.89
		4.5	285.90
		5	286.78
		5.5	287.56
		6	288.25
		6.5	288.88
		7	289.44
Methane + H_2_S (90–10)	0.616	3	286.46
		3.5	287.78
		4	288.90
		4.5	289.85
		5	290.69
		5.5	291.42
		6	292.06
		6.5	292.64
		7	293.15
Methane + H_2_S (85–15)	0.647	3	289.31
		3.5	290.61
		4	291.69
		4.5	292.61
		5	293.41
		5.5	294.11
		6	294.72
		6.5	295.26
		7	295.74
Methane + H_2_S (80–20)	0.678	3	291.49
		3.5	292.76
		4	293.82
		4.5	294.72
		5	295.49
		5.5	296.16
		6	296.74
		6.5	297.25
		7	297.71
Methane + H_2_S (75–25)	0.710	3	293.26
		3.5	294.50
		4	295.54
		4.5	296.41
		5	297.16
		5.5	297.80
		6	298.36
		6.5	298.85
		7	299.27
Methane + H_2_S (70–30)	0.741	3	294.74
		3.5	295.96
		4	296.97
		4.5	297.83
		5	298.55
		5.5	299.17
		6	299.72
		6.5	300.16
		7	300.56

**Table 4:** Data Points Generated by Simulation Using PVTSim Software for Carbon Dioxide Gas and Hydrogen Sulfide Gas Mixtures

Gas Sample	Gas Gravity	Pressure (MPa)	Temperature (K)
Carbon Dioxide	1.519	2	277.11
		2.5	278.87
		3	280.23
		3.5	281.32
		4	282.22
Carbon Dioxide + H_2_S (95–5)	1.502	2	283.01
		2.5	284.82
		3	286.23
		3.5	287.36
		4	288.32
Carbon Dioxide + H_2_S (90–10)	1.485	2	285.89
		2.5	287.72
		3	289.13
		3.5	290.26
		4	291.17
Carbon Dioxide + H_2_S (85–15)	1.468	2	287.99
		2.5	289.83
		3	291.25
		3.5	292.37
		4	293.28
Carbon Dioxide + H_2_S (80–20)	1.451	2	289.69
		2.5	291.53
		3	292.94
		3.5	294.06
		4	294.96
Carbon Dioxide + H_2_S (75–25)	1.434	2	291.13
		2.5	292.95
		3	294.36
		3.5	295.47
		4	296.37
Carbon Dioxide + H_2_S (70–30)	1.417	2	292.37
		2.5	294.19
		3	295.59
		3.5	296.69
		4	297.58
